# Thermodynamically consistent modeling of granular soils using physics-informed neural networks

**DOI:** 10.1038/s41598-025-12844-4

**Published:** 2025-07-28

**Authors:** Nazanin Irani, Mohammad Salimi, Torsten Wichtmann

**Affiliations:** Chair of Soil Mechanics, Foundation Engineering, and Environmental Geotechnics, Ruhr-University Bochum, Bochum, Germany

**Keywords:** Physics-informed neural networks, GINN, Thermodynamics laws, Constitutive modelling, Energy conservation, Civil engineering, Computer science, Applied mathematics

## Abstract

In recent years, data-driven approaches have gained considerable momentum in the scientific and engineering communities, owing to their capacity to extract complex patterns from high-dimensional data. Despite their potential, these approaches often require extensive high-quality datasets, may exhibit limited extrapolation capability beyond the training domain, and lack a rigorous foundation grounded in physical and thermodynamic principles. To overcome these limitations, physics-informed neural networks have been introduced, embedding governing equations directly into the learning process. Building upon this paradigm, this study presents a novel thermodynamically consistent constitutive model for granular soils, developed within the framework of geotechnically- and physics-informed neural networks (GINN). The model integrates physical laws with data-driven learning via a composite loss function. These include: (i) strictly non-negative material dissipation rate to ensure thermodynamic admissibility, (ii) an admissible range for the predicted stress state, and (iii) bounds on the predicted void ratio. The material dissipation rate is calculated using the total work input and a free energy potential expressed in terms of stress invariants. The model is validated against monotonic drained triaxial test data for specimens prepared with diverse initial void ratios and stress states. The model accurately simulates both the shear strength and dilative response of granular soil samples. Its predictive performance is further benchmarked against two widely adopted constitutive models from the literature, demonstrating comparable accuracy while maintaining consistency with thermodynamic laws.

## Introduction

The advent of big data analytics is actively transforming the field of material modeling, driving a shift from conventional methods reliant on limited experimental data to data-driven approaches powered by large-scale datasets. Building on the remarkable success of machine learning techniques in various geotechnical engineering applications-including the prediction of pile bearing capacity^1,2^, characterization of soil properties^3–5^, detection of cracks and leakage in tunnels^6,7^, slope stability analysis^8,9^, soil-structure interaction^10^, and urban planning initiatives^11^-there is growing interest in applying neural networks to the development of soil constitutive models. These emerging data-driven approaches aim to capture the full spectrum of material behavior by leveraging large and diverse datasets, thereby potentially eliminating the need for explicit analytical formulations. In contrast to physical models, which depend on prior knowledge of parameter relationships that are often unclear and must be carefully hand-engineered, deep learning methods extract feature representations exclusively from data without relying on predefined equations or assumptions^12^. A major challenge in developing purely data-driven models is their limited ability to extrapolate beyond the data on which they were trained^13,14^. In other words, these models often struggle to perform well in scenarios that differ from the training set. This limitation mainly stems from their inherent lack of a solid physical foundation^15^. When applied to soil constitutive modeling, this limitation presents significant practical challenges-particularly when artificial neural networks are used in boundary value problems involving real-world case studies. For example, consider the foundation of a wind turbine, where the subsoil is subjected to complex, multidirectional loading conditions induced by environmental forces such as wind, tides, ocean currents, wave action, and seismic events. These complex loading conditions may extend beyond the range of the training data, which could affect prediction accuracy and the reliability of design outcomes. To address the challenge of limited extrapolation, physics-informed neural networks offer a promising solution by integrating fundamental principles from physics and thermodynamics^15,16^. By embedding these physical laws, such models enhance predictive accuracy and reduce reliance on extensive training datasets^17,18^.

According to the thermodynamics laws, the rate of material dissipation must be non-negative, thereby ensuring energy conservation within the system. In a thermodynamically consistent framework, both stress and energy are reversible, and any closed strain cycle corresponds to a closed stress loop^19^. This, in turn, enables accurate predictions under closed strain paths and cyclic loading^20^. However, many constitutive equations commonly used in engineering practice violate the fundamental thermodynamic principles. Notably, numerical evidence has shown that such violations can lead to unsafe designs^21–23^, primarily due to the prediction of irreversible strain accumulation under repeated loading and unloading cycles^24–28^. This underscores the need for further development of thermodynamically consistent models that can overcome these challenges while ensuring safety and reliability. Conventional models mainly enforce granular thermodynamics through two scalar potentials, the dissipation rate and the free energy function^29–32^. Recent advancements have been incorporated to capture meso- to macro-scale mechanical mechanisms, including particle shape distribution^33^, sample preparation effects^34^, and soil anisotropy^35,36^, as well as elastoplastic coupling^23,37^. On the other hand, deep learning-based models can inherently enforce thermodynamic consistency by incorporating customized loss functions that penalize violations of energy dissipation^38,39^. These models provide enhanced flexibility in capturing heterogeneous, multiscale material behavior without relying on explicitly hand-crafted constitutive equations, thereby reducing the need for manual calibration while ensuring adherence to energy conservation principles. Moreover, the unsupervised identification of internal state variables-essential for describing the nonlinear, inelastic evolution of microstructure-is also achievable within this framework^40^. The use of GINN to develop thermodynamically admissible benchmark models paves the way for significant advancements, including the robust simulation of micro-to-macro scale responses and their implementation in boundary value problems via user-defined material subroutines.

This study introduces a novel thermodynamically consistent constitutive model for granular soils. The model combines fundamental granular thermodynamics with data-driven learning through a composite loss function that incorporates the following constraints: (i) a strictly non-negative dissipation rate, (ii) confinement of the predicted stress state within an admissible range, and (iii) prescribed bounds on the predicted void ratio. The dissipation rate is derived from the total work input and a free energy potential formulated in terms of stress invariants. Given the inherently non-linear and path-dependent nature of soil response, the model is designed to predict rate-based quantities rather than absolute values. The proposed framework is first validated against monotonic triaxial tests. Its predictive performance is then benchmarked against two established advanced constitutive models from the literature.

## General structure of the GINN

Mechanical constitutive models are used to describe the relationship between stress and strain for solids and geomaterials. These models take into account various multiscale mechanisms such as granular displacements, or mesoscale deformation localization, and express them in terms of homogenized (averaged) quantities. These quantities include stresses and strains, which are measured in a representative elementary volume. For uniaxial or multi-axial loading experiments on a variety of soils, nonlinear stress-stain curves at different stages of loading are reported by different researchers^41–46^. In continuum mechanics theory, the mechanical response of solid materials can be expressed through a generalized rate formulation:

1 $$\begin{aligned} \dot{\varvec{\sigma }} = f\left( \varvec{\sigma }, \zeta , {\varvec{\varepsilon }}, \dot{\varvec{\varepsilon }} \right) \end{aligned}$$

In this expression, $\varvec{\sigma }$ denotes the Cauchy stress tensor and ${\varvec{\varepsilon }}$ represents the strain tensor. The parameter $\zeta$ encompasses various internal state variables (for example, the void ratio observed in soil mechanics). The superposed dot denotes the material time derivative. Traditional constitutive models typically depend on highly nonlinear equations to characterize the stress-strain relationship. Alternatively, the same principles are leveraged here to develop a model for capturing this relationship within the GINN framework. The model operates in a strain-controlled manner, where at each step, the stress rate at $(n+1)$ is predicted based on the state variables at step *n* and the prescribed strain rate $\dot{\varvec{\varepsilon }}^{n}$. Accordingly, the inputs to the network should include the initial value of ${\varvec{\sigma }}^{n}$, ${\varvec{\varepsilon }}^{n}$, $\zeta ^{n}$, $\dot{\varvec{\varepsilon }}^{n}$, where $\zeta ^{n}$ denotes the internal state variables at step *n*. The network is then trained to predict the corresponding changes in stress and internal variables, i.e., the output consists of ${\dot{\varvec{\sigma }}^{n}, \dot{\zeta }^{n}}$. The proposed model is formulated within the triaxial stress space rather than the full tensorial domain, effectively reducing the dimensionality. This approach not only reduces the amount of training data required but also simplifies the input and output features that the neural network must process for each sample. Accordingly, stress (*p* & *q*) and strain invariants ($\varepsilon _v$ & $\varepsilon _q$) are employed in place of the full stress and strain tensors. The mean effective stress and deviatoric stress defined as $p=\sigma _{ii} / 3$ and $q=\sqrt{3{\sigma }^*_{ij}{\sigma }^*_{ij} / 2}$, respectively. Here, ${\sigma }^*_{ij} = \sigma _{ij} - \delta _{ij} p$ presents the deviatoric part of the effective stress tensor $\sigma _{ij}$ with $\delta _{ij}$ denoting the Kronecker delta. Similarly, the volumetric strain and deviatoric strain are defined as ${\varepsilon }_v = \varepsilon _{ii}$ and ${\varepsilon }_q = \sqrt{2{\varepsilon }^*_{ij}{\varepsilon }^*_{ij} / 3}$, where ${\varepsilon }^*_{ij}$ is the deviatoric part of the strain tensor.

### Selecting input and output features

**Fig. 1:**
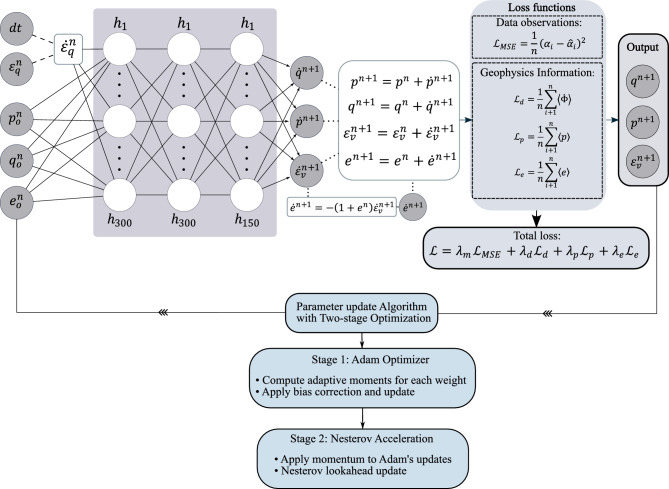
Architecture of the GINN: The inputs to the network consist of the time increment $\textrm{d}{t}$, the current level of deviatoric strain $\varepsilon ^n_q$, the initial stress components $p^n_0$ and $q^n_0$, and the initial void ratio $e^n_0$. The network outputs include the predicted stress state at the subsequent strain level, denoted as $p^{n+1}$ and $q^{n+1}$, along with the corresponding volumetric strain increment $\varepsilon ^{n+1}_v$. The void ratio at the new state, $e^{n+1}$, is computed explicitly using a predefined mathematical relationship, expressed as $e^{n+1} = -(1 + e^n)\varepsilon ^{n+1}_v$. In the schematic representation, quantities derived directly from analytical expressions are indicated by dashed lines, whereas solid lines correspond to predictions generated by artificial neurons within the network.

The model incorporates two fundamental state variables-stress and void ratio-which are essential for capturing the mechanical behavior of granular soils. The input parameters include the initial mean effective stress $p_0$, initial deviatoric stress $q_0$, and initial void ratio $e_0$. The model tracks the deviatoric strain path ${\varepsilon }_q$ by integrating its corresponding strain rate $\dot{\varepsilon }_q$. The unknown variables, including the stress state ($p^n$ & $q^n$) and volumetric changes at a predefined deviatoric strain level, are predicted using a fully connected deep neural network. Leveraging the general, material-independent analytical relationship between the rate of void ratio $\dot{e}^n$ and volumetric strain rate, the void ratio is subsequently computed directly from the predicted volumetric strain $\dot{e}^{n+1}= -(1+e^{n}) \dot{\varepsilon }_v^{n+1}$. Owing to the inherently non-linear and path-dependent behavior of soils, the model is designed to predict rate-based quantities rather than absolute values. Accordingly, the outputs consist of the rate of deviatoric stress $\dot{q}^{n+1}$, the rate of mean effective stress $\dot{p}^{n+1}$, and the rate of volumetric strain $\dot{\varepsilon }_v^{n+1}$. The predicted rates are subsequently integrated over the loading path to recover the total stress and strain responses. The architecture of GINN is depicted in Fig. 1 and will be discussed in further detail in the following sections.

### Ensuring thermodynamic consistency

Constitutive equations are required to satisfy the constraints of energy and entropy conservation, in accordance with the first and second law of thermodynamics. To characterize the constitutive behavior, two key potentials are required: the free energy function and either the dissipation or yield function, which are linked through a singular Legendre transformation^30,32,47^. This study focuses specifically on rate-independent materials under isothermal conditions. In this context, the rate of mechanical work $\dot{W}$ in an isothermal system is equal to the sum of the rate in Helmholtz free energy $\dot{\mathrm {\Psi }}$ and the dissipated energy $\dot{\Phi }$^48^:

2 $$\begin{aligned} \dot{W} = \dot{\mathrm {\Psi }} + \dot{\Phi }\,\,\,\,\,\,\,\,\,\, \text {with} \,\,\,\,\,\,\,\,\,\, \dot{\Phi }=\dot{W} - \dot{\mathrm {\Psi }} \ge 0 \end{aligned}$$

In this context, $\dot{\Phi }$ represents the increment of non-negative dissipated energy, while the rate of mechanical work is given by $\dot{W}= p \dot{\varepsilon _v} + q\dot{\varepsilon _q}$ holds. Energy potentials are typically formulated as functions of stress or strain invariants. In geotechnical engineering practice, it is generally more practical to approximate or determine the initial stress conditions within a soil profile than to accurately define the initial strain field. Consequently, expressing elastic moduli explicitly in terms of stress offers distinct advantages for geotechnical applications. In alignment with this rationale, a potential function defined in terms of stress invariants is pursued in this study. Irani et al.^49^ has performed a rigorous analysis to assess different energy potentials against mathematical conditions that ensures numerical stability. Furthermore, the application of the energy potentials that met these mathematical constraints was examined within the framework of bounding surface plasticity to model granular soil behavior. In this study, one of the proposed potentials from their work is adopted, which is expressed as follows:

3 $$\begin{aligned} \mathrm {\Psi }= & {\left[ \dfrac{p_a}{\left( 2-\chi \right) K_0} V^{\left( 2-\chi \right) } + \dfrac{q^2}{6p_a G_0} V^{\left( -2\chi \right) } \right] }\nonumber \,\,\,\, \text {with}\\ V= & \dfrac{1}{2p_a}\left( {3p} + \sqrt{{{p}^2}+\dfrac{2\chi K_0q^2}{3G_0}}\right) \end{aligned}$$

wherein $p_a$ is the atmospheric pressure, ${G}_0$ is a material parameter describing the initial shear modulus, $\chi$ is a material parameter characterizing the degree of homogeneity with values typically ranging from $0.5 \le \chi \le 0.9$^49^, and ${K}_0=0.75 {G}_0$ represents a reasonable assumption for granular soils^23^. The underlying physical law of energy conservation is expressed in Eq. 2. Accordingly, the GINN is constructed by predicting the rates of the state variables, which are subsequently updated at each time step. By modifying the loss function to incorporate both observational data and fundamental physical principles, the model replicates the soil response across varying levels of deviatoric strain. The rate of energy potential is then calculated through the following mathematical expression:

4 $$\begin{aligned} \dot{\mathrm {\Psi }} = \dfrac{\partial \Psi }{\partial p} \dot{p} + \dfrac{\partial \Psi }{\partial q} \dot{q} \end{aligned}$$

The first derivatives of the energy potential with respect to *p* and *q* are derived analytically within the model. To remain consistent with the laws of thermodynamics, the predicted stress state must always satisfy the inequality $\dot{\Phi }= p \dot{\varepsilon _v} + q\dot{\varepsilon _q} - \dot{\mathrm {\Psi }} \ge 0$.

### Definition of loss functions

Incorporating physical theory into deep learning entails systematically identifying theoretical constraints and integrating them into the architecture and training process of the learning model. This can be accomplished by formulating the dissipation energy and the admissible stress range as additional loss terms within the objective function. Considering a feed-forward artificial neural network with $k\ge 3$ layers, where *k* corresponds to a network comprising an input layer, $(k-2)$ hidden layers, and an output layer (the *k*-th layer). The signal within the network flows from layer (*k*-1) to layer (*k*) in accordance with:

5 $$\begin{aligned} \alpha ^{(k)}_j=\phi ^{(k)}\left( w^k_{jl}\alpha ^{(k-1)}_l+b^{(k)}_j\right) \end{aligned}$$

In this context, $\alpha ^{(k)}_j$ and $\phi ^{(k)}$ denote the output of node *j* and the activation function at layer (*k*), respectively. The weight between the *l*-th node in layer $(k-1)$ and the *j*-th node in layer (*k*) is given by $w^k_{jl}$ and $b^{(k)}_j$ is the bias of the *j*-th node in layer (*k*). The weights and biases can be adjusted through an iterative procedure to minimize the error between the target values and the predictions. This error is measured by a loss function, serving as a guide for parameter updates. Common loss functions include Mean Squared Error (MSE) for regression and Cross-entropy for classification tasks. Selecting an appropriate loss function plays a pivotal role in achieving efficiency in predictions. In this study, a total loss function *L* that consists of four terms representing the agreement between the network outputs and the training data is proposed:

6 $$\begin{aligned} L = \lambda _{m} L_{\textrm{MSE}} + \lambda _d L_{{d}} + \lambda _p L_{{p}} + \lambda _e L_{e} \end{aligned}$$

In this context, $L_{\textrm{MSE}}$ represents the data observation loss, while $L_d$, $L_p$, and $L_e$ denote geotechnically-informed loss functions. Loss components are weighted by the tuning parameters $\lambda _{\textrm{MSE}}$, $\lambda _d$, $\lambda _p$, and $\lambda _e$, such that $\lambda _{\textrm{MSE}} + \lambda _d + \lambda _p + \lambda _e = 1$ holds. These weights influence the model’s learning by balancing each loss component and can be methodically set or iteratively adjusted through tuning processes. The individual loss functions are defined as follows:

7 $$\begin{aligned} L_{\textrm{MSE}}= & \dfrac{1}{n}\sum _{i=1}^{n}({\alpha }_i -{\widehat{\alpha }}_i)^2 \end{aligned}$$

8 $$\begin{aligned} L_{d}= & \dfrac{1}{n}\sum _{i=1}^{n} \left\langle \dot{\Phi }\right\rangle \end{aligned}$$

9 $$\begin{aligned} L_{p}= & \dfrac{1}{n}\sum _{i=1}^{n}\left\langle p \right\rangle \end{aligned}$$

10 $$\begin{aligned} L_{e}= & \dfrac{1}{n}\sum _{i=1}^{n} \left\langle e \right\rangle \end{aligned}$$

with $\langle x \rangle = x$ for $x > 0$, and 0 otherwise. *n* is the number of data samples, $\alpha _i$ represents the actual target values, and $\widehat{\alpha }_i$ represents the predicted values (the final output of the network). $L_{d}$ adds limits for the model to predict non-negative energy dissipation rate in a thermodynamically consistent framework. $L_p$ and $L_e$ are losses that measure the error of the predicted negative effective stress and void ratio at any time *t*, respectively. A negative mean effective stress is physically unrealistic, as it implies tensile conditions that soils cannot sustain. Moreover, a negative void ratio is physically impossible because it implies that the volume of voids is less than zero. If $\alpha$ aligns with $\widehat{\alpha }_i$, and the assumed constraints are satisfied at all time points *t*, the total loss would be zero. In this study, equal values were chosen for the parameters, with $\lambda _{\text{MSE}} = \lambda _d= \lambda _p = \lambda _e = 0.25$. This choice is made for the sake of simplicity; however, as demonstrated in the evaluation section, adjusting these weights allows the learning procedure to be optimized with a simpler network architecture.

### Training and tuning hyperparameter

The process of updating the weights and biases, commonly known as the training process, is conducted on 50% of the available experimental dataset, which is defined as the training set. Once the GINN is trained, a test set, which is different from the training set, is utilized to examine the error of the network predictions. In this stage, an iterative evaluation and adjustment process was employed. An appropriate batch size of 30 was determined, and regularized weights were applied to maintain a consistent order of magnitude for the various quantities involved in the loss functions. The model architecture is a feed-forward neural network with three hidden layers: the first two layers contain 300 neurons each, and the third contains 150 neurons. The Rectified Linear Unit (ReLU) activation function is employed throughout the hidden layers to introduce non-linearity while avoiding the vanishing gradient problem common in deeper networks. To utilize the energy potential introduced in Eq. 3, two material constants must be specified: $G_0$ and $\chi$. Then, $K_0=0.75 G_0$ can be employed. Since these parameters have clear physical interpretations, they are directly assigned based on the properties of the material used in this study. An initial shear modulus of 150 MPa has been used for Karlsruhe Fine Sand (KFS)^49,50^. Furthermore, a value of $\chi = 0.5$ is considered, as it is considered a reasonable assumption for the degree of homogeneity in sands^49^. A learning rate of 0.005 and 600 training epochs are used. For optimization, the Adam optimizer with Nesterov’s accelerated gradient is employed, as also suggested by^14,51^. Adam is an adaptive learning rate optimization method that computes individual learning rates for each parameter by estimating first and second moments of the gradients. Specifically, it maintains exponentially decaying averages of past gradients (the first moment) and past squared gradients (the second moment), enabling adaptive step sizes that improve convergence stability and speed. To further enhance convergence performance, Nesterov’s accelerated gradient is applied as an additional momentum layer on top of Adam’s computed updates. In this approach, Adam first determines the parameter update direction and magnitude using its adaptive learning rates, then Nesterov acceleration uses momentum from previous iterations to anticipate the trajectory and make a corrective step that reduces oscillations and accelerates movement toward the optimum. This dual-layer optimization framework combines Adam’s adaptive learning capabilities with Nesterov’s momentum-based trajectory correction, providing enhanced convergence speed and stability in the complex parameter space of the proposed constitutive model, particularly effective for handling noisy gradients and avoiding local minima.

## Model assessment and validation

### Validation against experimental data

The drained triaxial experimental dataset of KFS performed by Irani et al.^46,52–55^ is employed for validation of the proposed model. KFS is classified as SP according to the Unified Soil Classification System (USCS). It contains virtually no fines ($<1\%$) and exhibits the following key characteristics: a critical state friction angle of $\varphi _c = 33.1^{\circ }$, determined as the average from multiple cone pluviation tests conducted on very loose specimens ($D_r \approx 0\%$) in dry conditions; a specific gravity of $G_s = 2.65$; minimum and maximum void ratios of $e_{\min } = 0.677$ and $e_{\max } = 1.054$, respectively; a mean grain diameter of $d_{50} = 0.14$ mm; and a uniformity coefficient of $c_u = d_{60}/d_{10} = 1.5$.

**Fig. 2:**
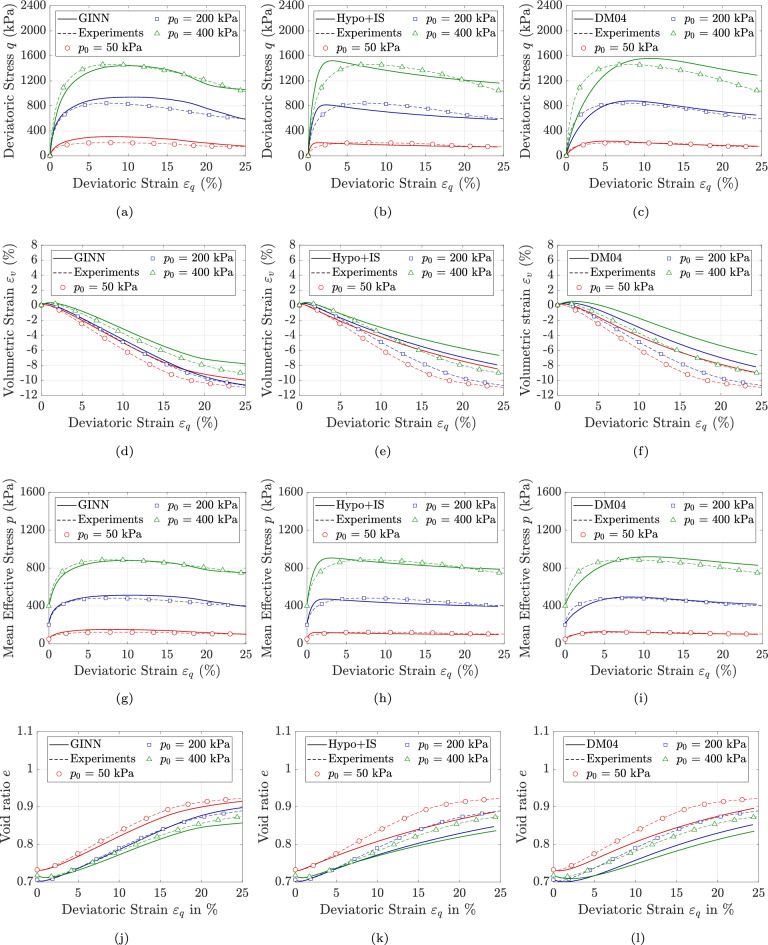
Model predictions versus experimental data for KFS under drained triaxial condition, with different initial stress levels of $p_0$ = 50, 200 & 400 kPa sheared with initial void ratios of $0.703 \le e_0 \le 0.731$ using (**a**, **d**, **g** & **j**): GINN model (**b**, **e**, **h** & **k**): Hypo+IS model, and (**c**, **f**, **i** & **l**): DM04 model.

In total, 25 drained triaxial compression tests were conducted on KFS specimens, covering a wide range of initial void ratios representative of loose, medium-dense, and dense states. The tests were performed under varying initial mean effective stress levels, ranging from 50 to 400 kPa. Among these, 13 tests were utilized for training the model, while the remaining 12 were reserved for validation. It is worth noting that this study only simulated the drained monotonic response of soils. To simulate different drainage conditions or samples subjected to multi-directional loading, additional data may be required to train the model. In this section, only the results from the test dataset are presented. Figure 2a, d, g & j illustrate the comparison between the GINN model predictions and the experimental results for the initial void ratio of $e_0 \approx 0.71$ and applied mean effective stress ranging from 50 kPa to 400 kPa. As shown in Fig. 2a, the model accurately captures the maximum deviatoric stress as well as the initial hardening response. Furthermore, Fig. 2d demonstrates the model’s ability to predict both contractive and dilative volumetric responses of KFS. In relation to Fig. 2g, the model is able to capture the evolution of mean effective stress under loading. It is worth mentioning that following the determination of $\dot{q}$, the model could, in principle, leverage the known stress path relationship from conventional triaxial tests, where the stress path follows a 1:3 slope. This would allow for a simplified formulation in which only the deviatoric stress rate needs to be predicted, while the mean effective stress could be directly computed using the relation $\dot{p} ={\dot{q}}/{3}$. Although this loading scenario reflects a conventional condition commonly employed in the evaluation of constitutive models, we deliberately chose not to hard-code this relationship into the model. Instead, the neural network is allowed to learn the underlying stress path behavior directly from the data. This decision highlights the model’s capacity to autonomously identify relevant patterns and disregard redundant or implicit inputs, such as those associated with conventional triaxial test constraints. As mentioned in the previous section, the model directly calculates the void ratio based on the predicted volumetric strain. Consequently, since the model effectively captures the evolution of volumetric strain, the resulting void ratio predictions in Fig. 2j show good agreement with experimental observations.

**Fig. 3:**
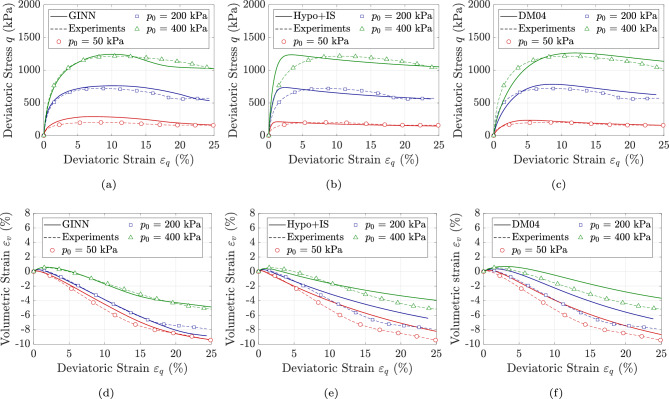
Model predictions versus experimental data for KFS under drained triaxial condition, with different initial stress levels of $p_0$ = 50, 200 & 400 kPa sheared with initial void ratios of $0.742 \le e_0 \le 0.789$ using (**a** & **d**): GINN model (**b** & **e**): Hypo+IS model, and (**c** & **f**): DM04 model.

**Fig. 4:**
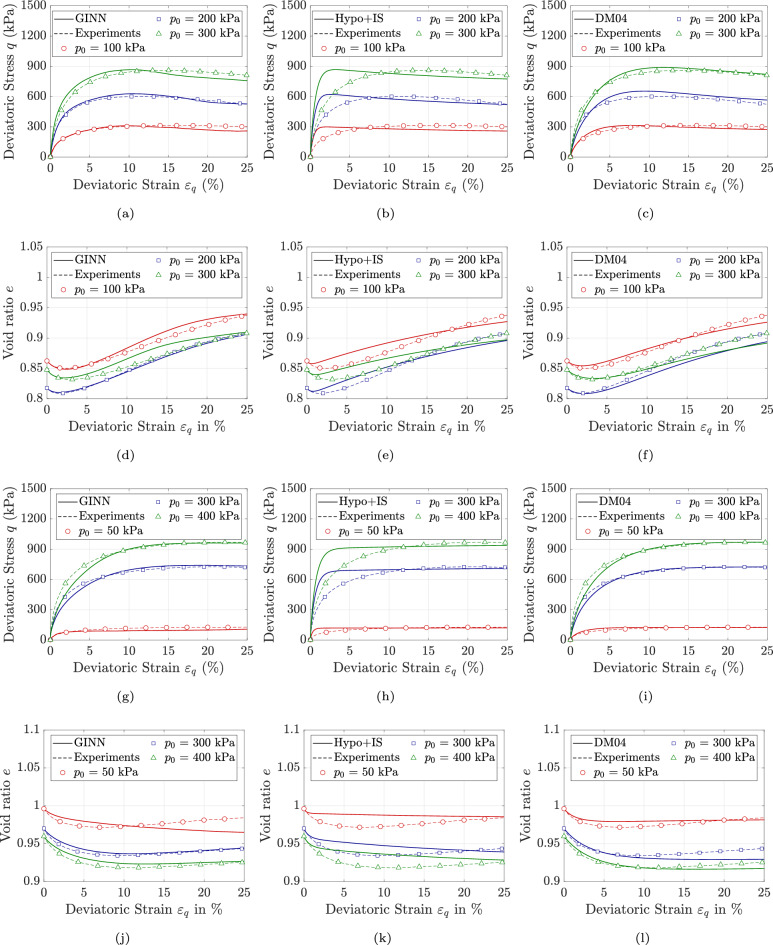
Model predictions versus experimental data for KFS under drained triaxial condition, with different initial stress levels of $p_0$ = 50, 100, 200 & 300 kPa sheared with initial void ratios of $0.809 \le e_0 \le 0.972$ using (**a**, **d**, **g** & **j**): GINN model (**b**, **e**, **h** & **k**): Hypo+IS model, and (**c**, **f**, **i** & **l**): DM04 model.

Figures 3a & d and 4a, d, g & j illustrate the performance of the GINN model in predicting soil response across a wide range of initial void ratios and stress states. For the sake of brevity, the results are illustrated only in the $q-p$ and $\epsilon _v-\epsilon _q$ planes. The comparisons between model predictions and experimental results show satisfactory agreement. Overall, the thermodynamically consistent formulation of the model, combined with its path-dependent integration scheme, enables it not only to represent the material behavior in an energy-conservative frame but also to learn effectively from a relatively limited dataset. It is worth noting that nearly 50 percent of the data is used solely for training, and the use of a large number of test samples for training was intentionally avoided. In particular, the GINN enhances the original loss function-which traditionally minimizes the weights and biases of the neural network-by incorporating physical principles as additional constraints.

### Comparative analysis with DM04 and Hypo+IS models

The predictive performance of the proposed model is benchmarked against two state-of-the-art and widely recognized constitutive frameworks. The first is the hypoplastic model for sands developed by Wolffersdorff^56^, subsequently enhanced through the incorporation of intergranular strain effects by Niemunis and Herle^57^; this enhanced formulation is hereafter referred to as HP+IS (Hypoplasticity with Inter-granular Strain). In general, hypoplasticity is a constitutive framework for modeling the non-linear, path-dependent behavior of soils without decomposing strain into elastic and plastic domains. It describes the stress-strain relationship through a single rate equation that directly relates the stress rate to the current stress, strain rate, and void ratio, enabling the capture of key aspects such as dilatancy, stiffness degradation, and loading-unloading response of soils. The second constitutive model is the bounding surface plasticity approach introduced by Dafalias and Manzari^58^, which explicitly accounts for fabric and its influence on dilatancy under loading reversal; this model is hereafter referred to as DM04 (Dafalias and Manzari 2004). The DM04 model is a state-dependent constitutive model grounded in critical state soil mechanics. It utilizes the state parameter introduced by Been and Jefferies^59^ to efficiently distinguish between dense and loose soil states. By incorporating a fabric-dilatancy deviator and a kinematic hardening rule, the model effectively captures soil behavior and has been successfully applied to boundary value problems. The successful and accurate predictions of these models in capturing soil response-both in element-level tests^60,61^ and finite element simulations^62^-have been well documented in the literature. As a result, they are employed as benchmarks for comparison with the proposed GINN. The simulations using the HP+IS model are illustrated in Fig. 2b, e, h & k, Fig. 3b & e and Fig. 4b, e, h & k, while the simulations for the DM04 model are shown in Fig. 2c, f, i & l, Fig. 3c & f and Fig. 4c, f, i & l.

For Hypo+IS, the material parameters calibrated by Wichtmann et al.^50^ are employed. Calibration of the DM04 model is also conducted based on KFS data. The parameters for HP+IS and DM04 are listed in Table 1 and Table 2, respectively. According to the results in Figs.( 2, 3 & 4), the proposed model demonstrates predictive capabilities comparable to those of the two other well-established advanced models in the literature, while maintaining a fully thermodynamically consistent framework. Moreover, the proposed model eliminates the need for parameter calibration, thereby significantly improving its ease of implementation and applicability in practical scenarios. Although the performance of the model has been evaluated at the element-level tests (i.e., at a single Gauss point), its effectiveness in solving boundary value problems requires further investigation. To this end, the model will be implemented in finite element codes as a user-defined material subroutine to address boundary value problems. However, this task lies beyond the scope of the present study and will be pursued in future work.

**Table 1:** Parameters of the HP+IS model for KFS reported by Wichtmann et al.^50^.

$\varphi _c$	$h_s$	*n*	$e_{d0}$	$e_{c0}$	$e_{i0}$	$\alpha$	$\beta$	*R*	$m_{R}$	$m_{T}$	$\beta _{r}$	$\chi$
${[}^{\circ }{]}$	[MPa]	[-]	[-]	[-]	[-]	[-]	[-]	[-]	[-]	[-]	[-]	[-]
33.1	4000	0.27	0.677	1.054	1.212	0.14	2.5	0.0001	2.2	1.1	0.1	5.5

**Table 2:** Parameters of the DM04 model used for the simulations.

$G_0$	$\nu$	$M_{\text {c}}$	*c*	$\lambda _{\text {c}}$	$e_{\text {c}}$	$\xi$	*m*	$h_0$	$c_h$	$n^b$	$A_0$	$n^d$	$z_{\max }$	$c_z$
[-]	[-]	[-]	[-]	[-]	[-]	[-]	[-]	[-]	[-]	[-]	[-]	[-]	[-]	[-]
50	0.25	1.34	0.71	0.122	1.103	0.205	0.05	5.5	0.75	1.5	1.0	1.6	20	10000

### Impact of loss function weighting on GINN performance

**Fig. 5:**
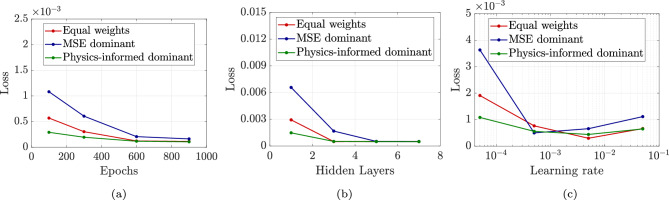
Variation in loss with respect to (**a**) number of epochs, (**b**) number of hidden layers, and (**c**) learning rate, for three distinct loss weighting strategies: equal weights ($\lambda _{\textrm{MSE}} = \lambda _d = \lambda _p = \lambda _e = 0.25$), MSE-dominant ($\lambda _{\textrm{MSE}} = 0.7$, $\lambda _d = \lambda _p = \lambda _e = 0.1$), and physics-informed dominant ($\lambda _{\textrm{MSE}} = 0.1$, $\lambda _d = \lambda _p = \lambda _e = 0.3$).

To investigate the effect of different weight values on model performance, a series of simulations is conducted by systematically varying the $\lambda$ parameters in the loss function of GINN. Three distinct weighting strategies are examined: (i) Equal weights ($\lambda _{\textrm{MSE}} = \lambda _d = \lambda _p = \lambda _e = 0.25$), providing balanced emphasis across all loss components; (ii) MSE dominant ($\lambda _{\textrm{MSE}} = 0.7$, $\lambda _d = \lambda _p = \lambda _e = 0.1$), prioritizing data-driven fitting over physics constraints; and (iii) Physics-informed dominant ($\lambda _{\textrm{MSE}} = 0.1$, $\lambda _d = \lambda _p = \lambda _e = 0.3$), emphasizing geotechnical constraints over pure data fitting. For each weighting strategy, the influence of training duration (number of epochs), network architecture (number of hidden layers), and learning rate on the total loss was assessed and is presented in Fig. 5. The analysis in Fig. 5(a & b) indicates that MSE-dominant configurations exhibit rapid initial convergence but require increased network complexity, with notable performance degradation observed when the number of hidden layers is insufficient. In contrast, physics-informed dominant weighting demonstrates superior stability across all tested conditions, maintaining consistent performance regardless of network depth or training duration. This suggests that incorporating physics-based constraints provides effective regularization. The equal weights strategy consistently yields intermediate performance, reflecting a balanced trade-off between objectives and offering practical advantages for robust model deployment. According to Fig. 5c, the loss for both the equal weights and physics-informed dominant configurations converges optimally at a learning rate of 0.005, whereas the MSE-dominant configuration displays greater sensitivity to this parameter. It should be noted that the presented results pertain specifically to triaxial drained loading conditions; under multidirectional loading or alternative drainage scenarios, convergence behavior may differ.

## Conclusion

This study introduces a novel, thermodynamically consistent constitutive model for granular soils, developed within the GINN framework. The model is trained using two scalar potential functions, specifically the free energy and the material dissipation rate. To address the inherently nonlinear and path-dependent nature of soil response, rate-based quantities are predicted by the model rather than absolute values. Crucially, fundamental physical constraints are embedded within the learning process, including a strictly positive material dissipation rate and admissible bounds for both the predicted stress state and void ratio. This ensures stiffness compatibility with granular thermodynamics. Furthermore, the energy-conserving formulation guarantees the reversibility of stress and energy during closed strain cycles. In this framework, the material dissipation rate is calculated from the total work input and a free energy potential expressed in terms of stress invariants. The model is initially validated against triaxial experimental data, with only 50% of the available dataset used for training and the remainder reserved for testing. Following validation at the element-level tests, the predictive capability of the model is benchmarked against two widely used advanced constitutive models from the literature. The results demonstrate that the proposed model achieves comparable accuracy while inherently conserving energy and reducing the number of parameters to be calibrated.

## Data Availability

The datasets and materials used and analyzed during the current study are available from the corresponding author upon request.
